# Spatial variation of phytoplankton composition, biovolume, and resulting microcystin concentrations in the Nyanza Gulf (Lake Victoria, Kenya)

**DOI:** 10.1007/s10750-012-1062-8

**Published:** 2012-03-14

**Authors:** L. Sitoki, R. Kurmayer, E. Rott

**Affiliations:** 1 Kenya Marine and Fisheries Research Institute, P.O. Box 1881, Kisumu, Kenya; 2 Institute of Botany, University of Innsbruck, Sternwartestraße 15, 6020 Innsbruck, Austria; 3 Institute for Limnology, Austrian Academy of Sciences, Mondseestraße 9, 5310 Mondsee, Austria

**Keywords:** Harmful algal blooms, Horizontal distribution, *Microcystis*, Seasonality, Toxicity, Health risk

## Abstract

The Nyanza Gulf is a large shallow bay of Lake Victoria suffering from eutrophication by human activities. In order to characterize the harmful algal bloom formation as a consequence of eutrophication, both spatially and seasonally, environmental conditions, phytoplankton community composition, and microcystin (MC) concentrations were investigated monthly from Kisumu Bay, and bimonthly from the center of the gulf, as well as quarterly from the Rusinga Channel and the main basin of Lake Victoria between July 2008 and September 2009. The sites located in Kisumu Bay and the central gulf were most strongly affected by eutrophication, including increased nutrient concentrations and phytoplankton growth. More than 90% of the samples obtained from the gulf were dominated by cyanobacteria, whereas diatoms only dominated in the samples obtained from Rusinga Channel and the main lake. In general, *Microcystis* accounted for the largest part (>50–90%) of cyanobacterial biovolume. MCs were found in 35 (54%) out of 65 samples and were detected throughout the study period in the gulf, but only in two out of eight samples from the Rusinga Channel and the main lake. A significant linear relationship between *Microcystis* biovolume and MC concentration was observed (*n* = 65, *R* ^2^ = 0.88, *P* < 0.001). The highest MC concentrations were recorded in Kisumu Bay between November and March (max. 81 μg l^−1^) when *Microcystis* showed max. biovolume (18 mm^3^ l^−1^ in November 2008). The results suggest that seasonal variability did not outweigh the spatial differences in phytoplankton composition and MC production, which is seasonally persistent in Kisumu Bay.

**Electronic supplementary material:**

The online version of this article (doi:10.1007/s10750-012-1062-8) contains supplementary material, which is available to authorized users.

## Introduction

During the last half century, Lake Victoria has undergone drastic changes in its water quality and biota, which have been attributed to eutrophication and the introduction of exotic species (Hecky, 1993; Verschuren et al., 2002). Pollution (nutrient loading) into the lake has increased from both diffuse and point-sources and exerts considerable impact especially on the near-shore areas (Hecky et al., 2010). In addition, fisheries production influenced the trophic status of the lake by the introduction of the piscivorous Nile Perch. The signs of eutrophication were not visible until the 1980s when, for the first time, extensive phytoplankton blooms, massive fish kills, and near-shore belts of water hyacinth *Eichhornia crassipes* (Mart.) Solms were observed (Ochumba & Kibaara, 1989; Ochumba, 1990).

Between January and March 2004, the persistence of massive phytoplankton blooms in Kisumu Bay of the Nyanza Gulf resulted in a temporary shutdown of the drinking water supply from the lake. Although the algal blooms were clearly visible, the type of contamination and the underlying causes were not investigated. Scientific evidence of nutrient enrichment in the Nyanza Gulf has been reported by Lung’ayia et al. (2001) and Gikuma-Njuru & Hecky (2005). Because of a continuously increasing wastewater discharge mostly from the East and South-east sides of the gulf (Calamari et al., 1995), trophic conditions further increased across the gulf (Gikuma-Njuru & Hecky, 2005). On the other hand, in other gulfs, the concentration of phytoplankton biovolume was found to be relatively low when compared with the observed nutrient concentrations (e.g. Sekadende et al., 2005; Haande et al., 2011). For the Nyanza Gulf, it has been concluded that the high mineral turbidity in the gulf reduces light availability and, therefore, limits algal abundance (Gikuma-Njuru & Hecky, 2005). In other bays of Lake Victoria, significant dilution effects because of massive water exchange with the main basin have been invoked to explain the relatively low phytoplankton biovolume (Haande et al., 2011). These authors concluded that surface seiches might cause considerable daily water exchange in Murchison Bay (Uganda) and could explain why the increase in total phytoplankton biomass in consequence of nutrient enrichment is not detectable. Similarly, dilution effects from the main basin due to the strong seasonal SE winds were invoked to explain the low phytoplankton abundance in the Mwanza Gulf (Tanzania), although near-shore nutrient enrichment was clearly visible (Sekadende et al., 2005). Consequently, the sensitivity of near-shore areas to eutrophication also depends on the connectivity to the main basin, which is variable both on a spatial and a seasonal scale. It is well known that wind directions are subject to seasonal change, i.e., generally dominant east to south-east during the dry season but west or north-west during the wet season. In the main basin of Lake Victoria, during the dry period (June to September and December to February), the water column increases in stability causing a reduced daily vertical mixing (Hecky, 1993). These physical changes have a significant effect on phytoplankton community composition, i.e., favoring the occurrence of buoyant cyanobacteria (Kling et al., 2001).

**Fig. 1:**
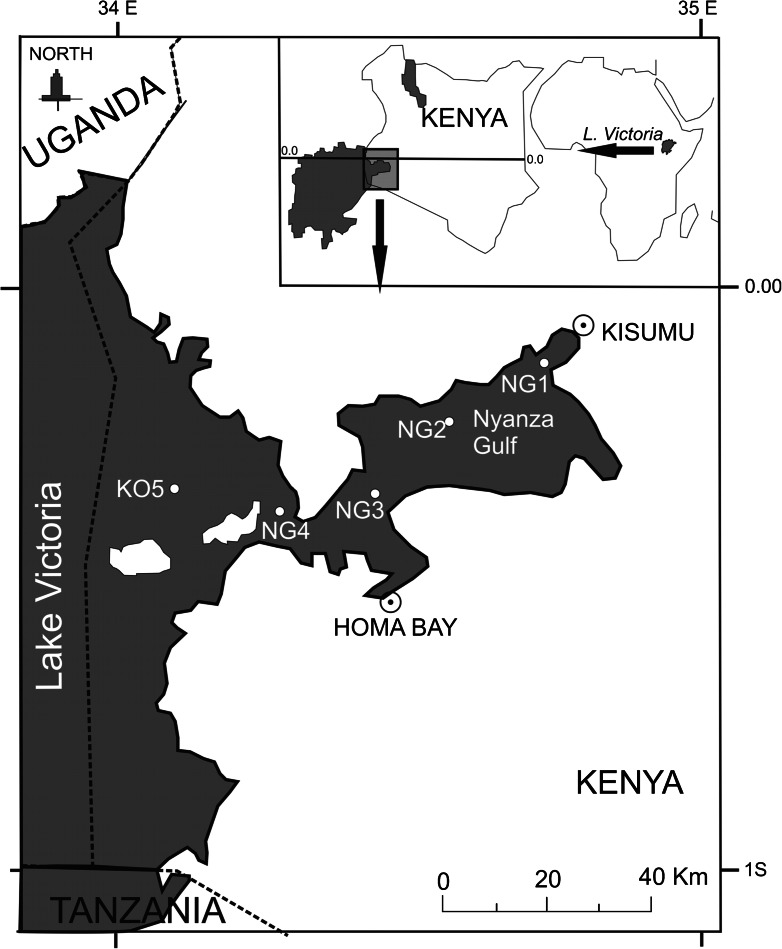
Map of Lake Victoria, Kenya sector, showing Nyanza Gulf and the locations of the five sampling stations (NG1, NG2, NG3, NG4, KO5)

The Nyanza Gulf is situated on the NE corner of Lake Victoria close to the equator and is one of the largest bays of L. Victoria (1,400 km^2^, mean depth 7 m, max. depth 30 m). The gulf has an irregular shoreline extending with its longest axis from SW to NE (Fig. 1), showing a pronounced spatial variation in the concentration of macronutrients, such as soluble reactive phosphorus (SRP), nitrate (NO_3_–N), and soluble reactive silica (SRSi) (Gikuma-Njuru & Hecky, 2005). Recently, hydrodynamic modeling revealed high horizontal dispersion rates and a strong connectivity with the main basin in the western part of the Nyanza Gulf (Okely et al., 2010). In contrast, in the eastern part of the gulf, the horizontal dispersion rates were much lower, and satellite images revealed significant sediment loading from inflow waters. Consequently, the high turbidity may actually prevent further phytoplankton growth in the eastern part of the Gulf, even under further increased nutrient enrichment conditions.

The aim of the study was to investigate the spatial and seasonal variabilities of eutrophication in the Nyanza Gulf both with regard to nutrient enrichment, phytoplankton composition, and the concentration of the hepatotoxin microcystin (MC). The typical climate at the Nyanza Gulf is characterized by two wet seasons, i.e., from March to May and October to December. However, in the year of this study (in 2008), the period July–September was exceptionally wet. While the spatial–temporal variability of the phytoplankton composition has been studied previously (Gikuma-Njuru & Hecky, 2005), the concentration of MC has never been recorded in a systematic manner. Several genera of cyanobacteria commonly occurring in Lake Victoria (*Anabaena, Microcystis*) have the potential to produce MC, which are the most widespread cyanotoxins with over 80 structural variants (Welker & von Döhren, 2006). Krienitz et al. (2002) reported MC production from surface water in Kisumu Bay, which was probably the first report on cyanotoxin occurrence from Lake Victoria. In the meantime, MC has been reported from the Mwanza Gulf, Tanzania (Sekadende et al., 2005), and several times from Uganda (Haande et al., 2007; Okello et al., 2010a, b; Semyalo et al., 2010). In the study of Krienitz et al. (2002), MC concentrations (<1 μg l^−1^) were reported during an *Anabaena* bloom (*Anabaena* spp. >90% of the phytoplankton biovolume with 8 × 10^5^ cells ml^−1^). The same authors stated that it was unclear as to whether the MCs that were detected could be attributed to all the cyanobacterial species found and the identification of the responsible MC-producing species requires clarification. In contrast, the results from several Ugandan waters including Lake Victoria showed that *Microcystis* was the only MC producer (Okello et al., 2010a, b). At the current state, it is necessary to clarify as to which cyanobacterial taxa are responsible for MC production in the Nyanza Gulf and which areas of the Nyanza Gulf are most severely affected. We assume that this information is valuable to guide a more safe water use not only for the water supply to the citizens of the town of Kisumu (1 million inhabitants) but also for the population in the neighborhoods that use the lake water directly.

## Materials and methods

### Study area and sampling

Between July 2008 and September 2009, site NG1 (max. depth 3.5 m), which was located most closely to Kisumu, was sampled monthly (Fig. 1). The other sites, located in the center of the gulf (NG2, 3), were sampled bimonthly, whereas the station in the Rusinga Channel (NG4) and in the main basin of Lake Victoria (KO5) were sampled quarterly. The maximum depths were 5, 10, 33, and 45 m at NG2, NG3, NG4, and KO5, respectively. In situ measurements of water temperature and conductivity were made with a submersible Conductivity–Temperature–Depth profiling system (CTD, Sea-bird Electronics^®^, Sea Cat SBE 19). These variables were recorded every 2 s when lowering the CTD from approximately 0.4 m (subsurface) to 0.4 m above the sediment. For each parameter, an average from the upper 10 m was calculated. Transparency was measured with a Secchi disk (Ø = 20 cm) painted black and white by taking the average of the depth at the disappearance and that of the reappearance of the disk in water.

Water samples were collected using a 3-l horizontal van Dorn sampler. A depth integrated sample was obtained by mixing the samples taken from every meter through the water column at stations NG1–NG3. At NG4 and KO5, only the upper 10 m of the water column were depth integrated. For phytoplankton analysis, an aliquot of the mixed water sample was fixed with Lugol’s solution. For nutrient analysis, pre-rinsed polyethylene bottles were filled with depth integrated samples and kept in an icebox for later analysis (usually within 24–72 h). In order to preserve the samples for later analysis, a few drops of 1 M HCl were added to induce pH 2 (Eaton et al., 2005). Although water samples from NG1 typically were processed and analyzed within 12 h (without adding HCl), the acidification of the samples from other sites with HCl probably led to the overestimation of the dissolved reactive nutrients, especially SRP. For MC analysis, 50–1,000 ml were filtered through glass fiber filters (Whatman GFC, Kent, UK) directly in the field using a vacuum pump. The filters were dried immediately in an oven at 45°C for 48 h and stored in the freezer at −18°C.

### Nutrient analysis

Soluble reactive phosphorous, nitrate nitrogen (NO_3_–N), and ammonia (NH_4_–N) were determined from the filtrate (Whatman GFC) using the ammonium molybdate method (Wetzel & Likens, 2000), sodium salicylate method (Müller & Wiedemann, 1955) and the indophenol blue method (Krom, 1982), respectively. Total phosphorus (TP) and total nitrogen (TN) were determined as SRP and NO_2_–N, respectively, from the unfiltered water sample subsequent to persulfate digestion (determination of NO_2_–N was made after passing the sample through a cadmium reduction column). Soluble reactive silica (SiO_2_) was determined as yellow molybdate-silicic acid (Wetzel & Likens, 2000).

### Phytoplankton analysis

Phytoplankton was identified and counted at 400× using an inverted microscope following the method of Utermöhl (1958). For each taxon, the cell length and width were measured from a minimum of 20 randomly selected specimens. Biovolume was calculated using geometric approximations (Rott, 1981; Wetzel & Likens, 2000). *Microcystis* colonies were measured at 40× magnification. The density of the cells per unit of the projection area of the colony was estimated using a higher magnification (400×). The depth of each colony was estimated by focusing on the top and bottom of the colony using the fine adjustment of the microscope. The following taxonomic literature was used for the identification of Cyanobacteria: Komárek & Anagnostidis (1998), Komárek et al. (2002), Komárek & Anagnostidis (2005). The eukaryotic algae taxonomic literature included Huber-Pestalozzi (1942) and Krammer & Lange-Bertalot (1988, 1991) for diatoms, Komárek & Fott (1983) for Chlorococcales and Huber-Pestalozzi (1950) for Cryptophyta and Dinophyta, and Talling (1987) for Zygnematophyceae. A detailed description of all phytoplankton species can be found in Sitoki (2010). Species diversity was expressed by means of the Shannon–Wiener diversity index (*H*).

### Microcystin analysis

Two sets of samples were analyzed: *Microcystis* strains and field samples. Sixteen strains of the genus *Microcystis* were isolated from NG1 and NG3 by streaking on an agar plate as described (Rippka, 1988). The strains were grown in the BG11 medium in a Heraeus culture chamber (25°C, 40 μmol m^−2^ s^−1^, 16:8 h light–dark cycle). Strains were harvested by filtering in the same way as the field samples. For the extraction of MCs from filters, 75% (w/v) methanol was used as described (Fastner et al., 1999). MC analysis was performed using HPLC–DAD with acetonitrile (0.05% trifluoroacetic acid) and Millipore water as solvents on a LiChrospher^®^ 100 RP – 18e (5 μm), LiChroCART^®^ 250-4 cartridge system using an HP 1100 ChemStation (Lawton et al., 1994, Kurmayer et al., 2003). MCs were identified by their retention time and characteristic UV absorption spectra. MCs were quantified at 240 nm, and the concentrations of all the MC variants were determined as MC-LR equivalents from the regression equation obtained using [D-MeAsp, D-Mdha]-MC-LR (Cyanobiotech GmbH, Berlin, Germany). HPLC fractions identified as MC were collected manually and analyzed by matrix-assisted laser desorption/ionization time-of-flight mass spectrometry (MALDI-TOF MS), (PerSeptive BioSystems, Framingham MS, USA) as described by Erhard et al. (1997). MCs are reported in volumetric units (μg l^−1^) and in cellular content (fg cell^−1^ or μg mm^−3^ of *Microcystis*).

### Statistical analysis

In order to determine the dependence of phytoplankton species abundance on the environmental parameters across sampling stations NG1–KO5, Canonical Correspondence Analysis (CCA) was performed. However, it was not possible to relate any of the environmental variables (Tables 1, 2) to the phytoplankton community composition by CCA with statistical significance. Multiple linear regression analysis was used to test the relationship between the *Microcystis* spp. biovolume and proportion and the influence of the nine environmental variables as used for CCA. A forward stepwise analysis was performed to select the independent variable for inclusion, which makes the most significant unique (or additional) contribution to the prediction of the data. The data were log transformed to fulfill the assumptions of normality and constant variance. The Durbin–Watson statistic was 2.4 (*Microcystis* biovolume) versus 1.8 (*Microcystis* proportion), implying that the residues of the regression were not related. The *F* value to enter the respective model was set as the default (*P* < 0.05) using SPSS 15.0 for Windows (SPSS GmbH Software, Munich, Germany).

**Table 1:** Temporal variation of environmental parameters at nearshore (site NG1)

Date	Temp (μg °C)	Cond (μS cm^−1^)	Secchi (m)	SRP (μg l^−1^)	TP (μg l^−1^)	SiO_2_ (mg l^−1^)	NO_3_–N (μg l^−1^)	NH_4_–N (μg l^−1^)	TN (μg l^−1^)	TN:TP (ratio)	MC (μg l^−1^)	Rain (mm)
Jul-08	24.9	181	0.30	108	398	33	40	192	1,713	4	na	107
Aug-08	27.2	131	0.20	96	378	48	41	209	1,813	5	na (274)^a^	127
Sep-08	27.7	167	0.25	95	316	32	35	207	1,551	5	na	94
*Oct-08*	*27.1*	*163*	*0.20*	*15*	*97*	*17*	*309*	*124*	*1,934*	*20*	*na (18.2)*	*150*
*Nov-08*	*27.5*	*156*	*0.20*	*18*	*80*	*24*	*195*	*138*	*1,934*	*24*	*80.7 (133)*	*206*
*Dec-08*	*27.1*	*163*	*0.20*	*79*	*191*	*23*	*5*	*148*	*839*	*4*	*72.5*	*143*
Jan-09	26.8	167	0.20	64	213	29	32	58	591	3	4.5	114
Feb-09	27.0	158	0.20	55	193	34	31	52	561	3	<0.01	50
*Mar-09*	*27.5*	*170*	*0.30*	*97*	*380*	*32*	*39*	*97*	*1,080*	*3*	*27.9*	*80*
*Apr-09*	*26.7*	*168*	*0.17*	*124*	*444*	*30*	*80*	*71*	*1,187*	*3*	*4.9*	*274*
*May-09*	*26.9*	*174*	*0.18*	*62*	*1,073*	*37*	*41*	*99*	*1,202*	*1*	*6.8*	*125*
Jun-09	25.8	166	0.22	119	199	25	22	125	1,197	6	4.0	28
Jul-09	26.2	176	0.20	102	233	32	76	145	1,706	7	6.9	31
Aug-09	26.3	167	0.20	9	291	29	25	182	na	na	3.5	77
Sep-09	26.7	171	0.20	74	84	30	8	114	411	5	8.1	46

*na* not analyzed

Italic values indicate the wet season. Values for temperature and conductivity were averages from the upper 10 m of the water column, whereas SRP, TP, SiO_2_, NO_3_–N, NH_4_–N, TN, and MC were analyzed from depth-integrated samples

^a^Values in parentheses refer to surface water samples

**Table 2:** Median and percentiles (25, 75%) of physico-chemical and biological parameters recorded at the five stations in Nyanza Gulf

Temp (°C)	Cond* (μS cm^−1^)	Secchi* (m)	SRP (μg l^−1^)	TP (μg l^−1^)	SiO_2_* (mg l^−1^)	NO_3_–N (μg l^−1^)	NH_4_–N* (μg l^−1^)	TN (mg l^−1^)	TN:TP (atomic ratio)	Biovolume (mm^3^ l^−1^)	MC* (μg l^−1^)	H*	*R*
*NG1*													
27.1	171	0.2	86	159	31	58	126	1.39	6.1	10.9	17.3	1.1	20
(26.5,27.5)	(163,174)^a^	(0.2,0.25)^a^	(46,100)	(82,307)	(27,32)^a^	(24,136)	(106,142)^a^	(0.75,1.82)	(3.9,15.8)	(6.2,15.1)	(5.6,59.2)^a^	(0.45,1.75)^a^	(15,23.5)
*NG2*													
25.4	160	0.35	68	220	28	42	105	1.41	8.1	10.8	14.2	1.2	16.5
(24.6,26.2)	(146,164)^a^	(0.3,0.45)^a,b^	(47,87)	(150,253)	(26,31)^a^	(24,59)	(70,111)^a,b^	(1.07,1.9)	(6.5,8.6)	(4.5,15)	(3.6,26.4)^a^	(0.85,1.6)^a^	(13,24.5)
*NG3*													
25.7	151	0.55	75	203	21	34	89	1.38	7.5	5.1	13.4	1.25	16
(25.2,26.6)	(148,152)^a,b^	(0.4,0.6)^a,b^	(39,88)	(138,266)	(21,21)^a,b^	(16,97)	(64,107)^a,b^	(1.11,1.73)	(6.5,8.1)	(3.9,8)	(5.6,20.3)^a^	(1.15,1.35)^a^	(14,19.5)
*NG4*													
25.9	117	0.95	31	199	9	9	18	1.33	10.3	6.4	0.01	2.1	18.5
(25.4,26.9)	(114,122)^b^	(0.8,1.35)^b^	(31,46)	(103,298)	(6,12)^b^	(7,96)	(12,21)^b^	(0.94,1.88)	(5.3,13)	(5.2,8.5)	(0.01,3.1)^a^	(1.8,2.25)^a^	(14,28.5)
*KO5*													
25.8	106	1.55	34	115	6	14	63	1.23	14.2	5.0	0.01	2.2	23.5
(25.4,26.8)	(101,112)^b^	(1.15,2.5)^b^	(26,38)	(84,188)	(5,11)^b^	(4,148)	(51,80)^a,b^	(0.73,1.63)	(8,15)	(4.2,6.4)	(0.01,0.5)^a^	(1.8,2.4)^a^	(19,26)

*H* Shannon–Wiener diversity index; *R* species richness

Asterisks mark parameters significantly different between sites (*P* < 0.05, Kruskal–Wallis one way ANOVA). Identical superscripts (a, b) indicate subgroups not significantly different at 0.05 (Tukey post hoc pairwise comparison, *n* = 4)

## Results

### Environmental conditions and phytoplankton composition in Kisumu Bay (NG1)

Throughout the study period, the temperature showed minor seasonal variations with the maxima observed during the wet seasons (Table 1). Water transparency was low and showed little seasonal variation (mean ± SD, 0.2 ± 0.03 m). Overall SRP concentrations were high (74 ± 37 μg l^−1^) and showed temporal variability with minima in October 2008 and July 2009. Nitrate (NO_3_–N) concentrations varied 60-fold between October 2008 (309 μg l^−1^, start of the wet season) and December 2008 (5 μg l^−1^, end of the wet season). The concentrations of NH_4_–N were generally higher than those of NO_3_–N with the minima occurring (52–99 μg l^−1^) from January to May 2009 and the maxima from August to September 2008 (>200 μg l^−1^). Reductions in TN concentrations were recorded from December to February, coinciding with the mats formed by the water hyacinth.

**Fig. 2:**
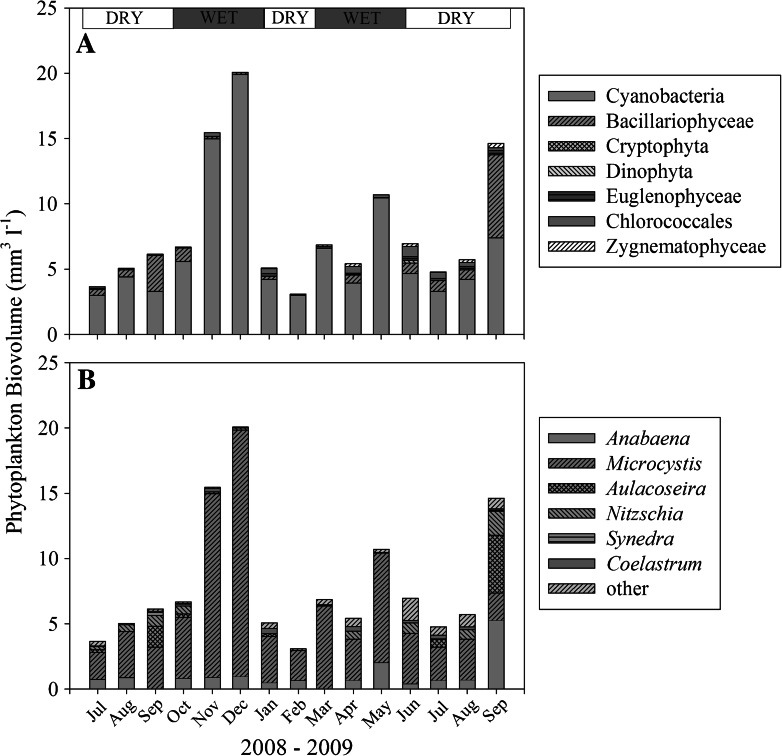
Absolute phytoplankton biovolume composition (mm^3^ l^−1^) assigned to phytoplankton classes or families (**A**) and phytoplankton genera (**B**) as recorded monthly from 2008 to 2009 at sampling station NG1. For the sake of clarity, only genera contributing >5% to total phytoplankton biovolume are shown. For species composition, see Supplement Table 1. *Top bar* indicates the periods of dry and wet seasons

Phytoplankton biovolume showed its maxima during the wet seasons (Nov–Dec 2008 and May 2009) and in September 2009 at the end of the dry season (Fig. 2). Cyanobacteria were the most dominant, contributing >50% to the total phytoplankton biovolume. Except for one seasonal maximum of *Anabaena* spp. (36%) in September 2009, *Microcystis* spp. constituted the major part (78 ± 16%) of the cyanobacteria. Within *Microcystis* spp. four species were observed according to morphological characters: *M. botrys* Teiling, *M. wesenbergii* Komárek, and the tropical species *M. panniformis* Komárek and *M. protocystis* Crow. Komárek et al. (2002) distinguished the two species from *M. aeruginosa* based on colony morphology and life cycle. *M. panniformis* formed spheroidal colonies with several adjacent parts appearing like large intestines. Macroscopic colonies of this species were cloudy, irregular, lobate, and disintegrated (cells were 4.5 ± 0.7 μm in diameter and 38 ± 9 μm^3^ in biovolume). *M. protocystis* differed from *M. aeruginosa* because of its obligatory sparse arrangement of cells in colonies, a characteristic that is observed in *M. aeruginosa* only in samples from benthic environments (Komárek et al., 2002). In addition, the species specific formation of special mucilaginous envelopes in individual cells (pseudovacuoles) of *M. protocystis* was observed. The colonies of *M. protocystis* were irregular, flat, and sometimes appear hollow (cells were 4.4 ± 0.7 μm in diameter and 40 ± 7 μm^3^ in biovolume). For subsequent calculations, all morphospecies were combined under *Microcystis* spp. Diatoms were abundant during the dry seasons in 2008 and 2009, and peaked in September. The seasonal succession of dominant diatom species was similar in both years: *Nitzschia* spp. occurred most frequently early in the dry season (June–July), whereas *Aulacoseira* sp. was the most abundant in the late dry season (September). From January to September 2009, there was a gradual increase in the abundance and diversity of Chlorococcales and Zygnematophyceae. Species diversity was significantly higher in the dry season (*H* = 1.79) than in the wet season (*H* = 0.88) in 2009 (*t* test, *n* = 3, *P* < 0.05).

### Environmental conditions and phytoplankton composition along the Nyanza Gulf (NG1-KO5)

**Fig. 3:**
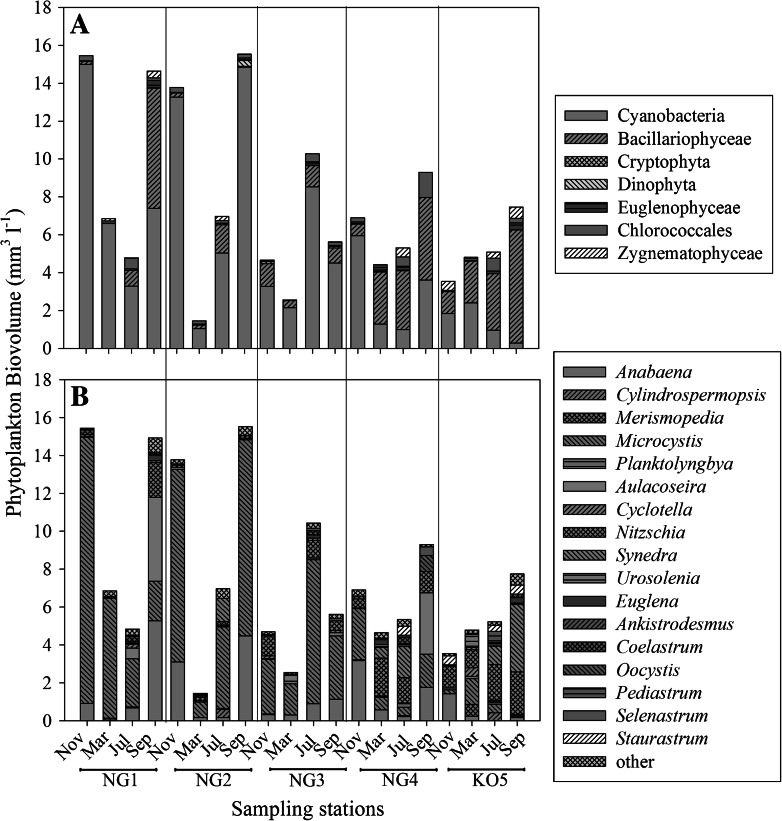
Absolute phytoplankton biovolume composition (mm^3^ l^−1^) assigned to phytoplankton classes or families (**A**) and phytoplankton genera (**B**) as recorded at four dates in 2008 (Nov) and in 2009 (Mar, Jul, and Sept.) for each sampling station (NG1, NG2, NG3, NG4, and KO5). For the sake of clarity, only genera contributing >5% to total phytoplankton biovolume are shown. For species composition, see Supplement Table 1

Dissolved nutrient fractions of P, N, and Si (SRP, NH_4_–N, and SiO_2_) as well as conductivity decreased from inshore to offshore (Table 2). In contrast, the Secchi depth significantly increased from NG1 (Kisumu Bay) to NG4 (Rusinga Channel) and to the main lake (KO5). In total, 101 phytoplankton species were identified and the phytoplankton composition showed striking differences along the transect. Cyanobacteria were the dominant group in 11 out of 12 samples from NG1 to NG3 and *Microcystis* spp. constituted >50% of the total algal biovolume (Fig. 3). *Anabaena* spp. was the most abundant at NG4 and KO5, i.e., contributed 46 and 40% at NG4 and KO5, respectively, each in November 2008. In contrast to the cyanobacteria, the diatoms increased in proportion from NG1 to NG3 to NG4 and KO5 and primarily comprised *Aulacoseira* spp., *Nitzschia* spp., *Synedra cunningtonii* G.S. West, and *Urosolenia victoriae* (Schröder) Rott & Kling. The Rusinga Channel and the main lake had significantly higher species diversity and evenness when compared with the inner gulf sites (Table 2). Algal biovolume showed its maxima at NG1 and NG2 and its minima at NG4 and KO5. The total phytoplankton biovolume was significantly correlated to the abundance of *Microcystis* spp. (*n* = 20, *R* ^2^ = 0.70, *P* < 0.008). It is concluded that seasonal variation did not outweigh the spatial difference in phytoplankton composition along the transect.

### Microcystin concentrations

Altogether, 65 field samples were analyzed, of which 35 samples were found to be MC positive (54%). In general, MCs were detected when *Microcystis* was dominant. The proportion of MC positive samples increased significantly when more than 10^6^ *Microcystis* cells were extracted and analyzed for MC (= 35 (73%) out of 48 samples). Six different variants of MCs could be undoubtedly identified (Table 3). MC-LR (M + H 995) and MC-YR (M + H 1045) were the most abundant variants comprising on average 50 ± 6 and 31 ± 5% of the total MC concentration, each. Unknown MC variants comprised 6.8 ± 3% of the total MC concentration. In total, 16 strains assigned to *M.* *panniformis* were isolated, and 12 strains (75%) were found to contain MC. In general, there was a close match between the proportion of MC structural variants recorded from the field samples and from the cultured *Microcystis* strains (Table 3). Nevertheless, within the strains, a large variation in the cellular MC content was observed (mean ± SE, 232 ± 49 fg cell^−1^ or 5.8 ± 1.2 μg mm^−3^ of biovolume, range 17–553 fg cell^−1^ or 0.4–13.8 μg mm^−3^ of biovolume).

**Table 3:** Frequency of occurrence (%) of MC variants and proportion in total MC (mean ± SE) as recorded from field samples (*n* = 35) and *Microcystis* strains (*n* = 12)

MC variant	M + H^+^	Structural variant	Retention (min)	Frequency		Proportion	
				Field	Strains	Field	Strains
MC1	1,024	[Asp3] MC-RR	14.1–14.2	11	0	5.4 ± 4	0
MC2	1,038	MC-RR	15.4	0	8	0	0.8 ± 0.8
MC3	1,063	[NMeSer7] MC-YR^a^	16.6–17.0	8	58	6.3 ± 3.8	5.2 ± 1.6
MC4	1,031	[Asp3] MC-YR	17.9–18.0	0	58	0.3 ± 0.3	32 ± 8.2
MC5	1,045	MC-YR	18.5–18.8	62	58	31 ± 52	17 ± 4.6
MC6	995	MC-LR	19.9–20.1	89	92	50 ± 6	32 ± 7
		Unknown	22.1–25.8	46	50	6.8 ± 3	14 ± 9

^a^Described by Okello et al. (2010b)

During *Microcystis* bloom formation, the highest MC concentrations were recorded, i.e., from surface scums occurring at NG1 in August and October 2008 MC concentrations of 274 and 133 μg l^−1^, respectively. MC concentrations gradually decreased from NG1 (Kisumu Bay) to NG2, NG3, and significantly lowest concentrations were recorded in Rusinga Channel (NG4) and KO5 (Table 2). At inshore (NG1), MCs were detected almost year-round with a maximum during the wet season (Table 1). Taking all the data together, the MC concentration was significantly correlated with *Microcystis* cell number: *y* = 0.0004*x* − 5.0487, or *Microcystis* biovolume: *y* = 10.498*x* − 5.0487 (*n* = 65, *R* ^2^ = 0.88, *P* < 0.001), where *y* is the MC concentration (μg l^−1^) and *x* is the *Microcystis* cell number (cells ml^−1^) or biovolume (mm^3^ l^−1^). In contrast, there was no correlation between the MC concentrations and the *Anabaena* cell number (*n* = 65, *R* ^2^ = 0.05, *P* = 0.98). Using multiple regression analysis, only the variable conductivity was included in the forward stepwise method, explaining the *Microcystis* biovolume: *y* = −11.501 + 5.502*x* (adjusted *R* ^2^ = 0.47, *n* = 20, *P* < 0.001) or *Microcystis* proportion: *y* = −9.451 + 5.104*x* (adjusted *R* ^2^ = 0.54), where *y* is the log_10_ *Microcystis* biovolume (mm^3^ l^−1^) or proportion and *x* the log_10_ conductivity (μS). It is concluded that the conductivity, which is reflecting at least partly nutrient input from the terrestrial runoff, can significantly predict *Microcystis* abundance that is further linearly related with the MC concentration.

## Discussion

### Nutrient enrichment in the Nyanza Gulf

The Nyanza Gulf is a semi-closed bay with a limited water exchange with the main basin (Calamari et al., 1995). The isolated situation distinguishes the Nyanza Gulf from the numerous more open bays where the impacts of eutrophication are probably diminished by dilution effects resulting from surface seiches (Haande et al., 2011). Spatial differences in conductivity along the main axis from the shallow areas of the Nyanza Gulf (NG1-3, Table 2) to the deeper sites of the central lake basin (NG4 and KO5) were reported previously (Gikuma-Njuru & Hecky, 2005; Hecky et al., 2010) and were confirmed in this study. As mentioned in those earlier studies, the evidence for eutrophication in the Nyanza Gulf comprises (1) a significant reduction in transparency, (2) shifts in phytoplankton composition from diatoms to cyanobacteria, and (3) increased macronutrient concentrations. In 2008, Gikuma-Njuru reported that the average TP concentration almost doubled from 68 μg l^−1^ (2000–2002, Gikuma-Njuru & Hecky, 2005) to 107 μg l^−1^ (2005–2006). During this study, TP concentration further significantly increased (on average 199 μg l^−1^, Table 2) suggesting that severe eutrophication occurred within a period of less than 10 years. However, the increase in TP is not matched with a similar increase in TN, which has changed from an average of 983 μg l^−1^ in 2000–2002 (Gikuma-Njuru & Hecky, 2005) to 1,356 μg l^−1^ in the present study. A large portion of this nutrient increase can be attributed to the allochthonous fluvial nutrient inputs from agricultural and urban areas in the catchment (Gikuma-Njuru, 2008; Hecky et al., 2010). The rapid increase in nutrient concentrations seems to be further enhanced by heavy rainfall (Hecky et al., 2010). Based on the average TP concentrations, the trophic classification system for tropical lakes (Salas & Martino, 1991) assigns the Nyanza Gulf to a hypereutrophic state. On the other hand the dissolved inorganic nitrogen (DIN) mainly NO_3_–N concentration is relatively low when compared with TN. This discrepancy between DIN and TN seems to be a common feature of many tropical water bodies. For example, in Lake Maracaibo, Venezuela nitrogen availability was found to be regulated by efficient diurnal nutrient (nitrogen) recycling based largely on dissolved organic nitrogen in spite of low DIN (Gardner et al., 1998).

### Functional groups of phytoplankton in the Nyanza Gulf

In accordance with the functional classification system (Reynolds, 2006), the following functional groups were recognized: (i) Group M, as represented by *Microcystis* spp. inhabiting shallow, daily mixed layers in eutrophic lakes. It is well known that this species tolerates high insolation but is sensitive to flushing and low total light availability (Paerl et al., 1983). This group M formed surface blooms (NG1–NG3) most of the year (Fig. 3); (ii) Group H, as represented by nitrogen-fixing *Anabaena* spp. was observed in high abundance only at NG4 (46%) and KO5 (40%) in November 2008, and at NG1 (36%) in September 2009. This group is believed to tolerate low DIN availability but is considered sensitive to mixing and poor light conditions; (iii) Groups C, P as represented by large centric diatoms (*Aulacoseira* spp.) that increased in abundance at NG1 and NG4 during the dry season (peak in September 2009); and (iv) Group N, thin and long diatoms (e.g., *Nitzschia* spp. and *Synedra cunningtonii*) found in highest abundances at KO5 at the end of the dry season (September 2009).

Cyanobacteria were dominant at the three sites within the gulf reaching from Kisumu to station NG3 covering a distance of 40 km, which corresponds to >50% of the total area of the shallower sector of the Nyanza Gulf. The persistent dominance of cyanobacteria was almost exclusively due to *Microcystis* spp. contributing >70% of cyanobacterial biovolume. According to Komárek et al. (2002) the dominating *Microcystis* morphospecies were classified into *M. panniformis* and *M. protocystis* rather than into *M.* *aeruginosa* (Kützing) Kützing and *M. flos*-*aquae* (Wittrok) Kirchner. In contrast, the occurrences of *M.* *aeruginosa* and *M.* *flos*-*aquae* in Nyanza Gulf have been reported previously (e.g., Lung’ayia et al., 2000). In earlier studies on L. Victoria, the morphospecies of *Microcystis* spp. were either assigned to *M.* *aeruginosa* (Kützing) Kützing (Haande et al., 2007) or *M.* *wesenbergii* and *M.* *aeruginosa* (Haande et al., 2011), or were not differentiated (e.g., Okello et al., 2010a, b). We cannot fully exclude the occurrence of *M. aeruginosa,* however, it is likely that it was not dominant during the period of this study. Since the morphospecies of *M.* *panniformis* and *M.* *protocystis* have been described relatively recently (Komárek et al., 2002) it is possible that colonies of *M.* *panniformis* have been described as *M. aeruginosa* earlier. Alternatively, it has been shown that morphological variation of *Microcystis* spp. can occur seasonally within the same waterbody which could be because of changes in *Microcystis* spp. genetic composition (e.g., Harada et al., 2001; White et al., 2003; Ozawa et al., 2005). The absolute dominance of *Microcystis* spp. is in striking contrast to the more diverse phytoplankton species composition in Murchison Bay in Uganda where besides *Microcystis* spp. (usually <30% of cyanobacteria biovolume) several other genera comprised significant portions of the cyanobacterial biovolume, i.e., *Anabaena* spp. (10%), *Aphanocapsa* spp. (20%), *Planktolyngbya circumcreta* G.S. West (10%), *Gomphosphaeria aponina* Kuetzing (10%), and *Merismopedia* spp. (15%), (Okello et al., 2010a; Haande et al., 2011).

In an environment with high phosphorus availability, nitrogen limitation can play a significant role in selecting dominant taxa (Reynolds, 2006). Nitrogen limitation has been considered the principal factor leading to the proliferation and dominance of heterocystous cyanobacteria in large parts of Lake Victoria (Kling et al., 2001) and in the Nyanza Gulf (Gikuma-Njuru & Hecky, 2005). The TN:TP ratio observed in the Nyanza Gulf corresponds closely to recent findings from highly eutrophic Ugandan shores (range 7–15 atomic ratio, Haande et al., 2011). In Kisumu Bay, neither the low TN:TP ratio nor the low DIN:SRP ratio can explain the dominance of the non-nitrogen fixing *Microcystis*. Here, and in other parts of the gulf (the deep locations of NG4 and KO5), it is possible that physical factors, such as the relationship of the euphotic zone to the mixing depth and/or variations in turbidity (organic and mineral seston) rather than TN:TP ratios, regulate phytoplankton composition. Indeed, it is likely that under turbid and polymictic conditions *Microcystis* has an advantage over *Anabaena* in shallow waters even under N-limiting conditions. When compared with *Anabaena*, *Microcystis* forms larger colonies (colonies of *Microcystis* >1 mm in diameter were observed). Because of buoyancy, the larger colonies can rise to the surface more quickly, and *Microcystis* would benefit from the increased light availability to a larger extent than *Anabaena*. In the shallow part of the gulf, mixing allows for sufficient light availability to sustain the growth of *Microcystis* over the extended periods even when the depth of the euphotic zone is much lower than the mixing depth (*Z*_eu_ ≪ *Z*_mix_). In contrast, the dominance of the nitrogen-fixing *Anabaena* is not favored by continuous mixing but requires temporarily stratified conditions, as observed offshore (Gikuma-Njuru & Hecky, 2005). The increasing dominance of diatoms in the Rusinga Channel and in the main lake station seems to be facilitated by a reduced turbidity resulting in the higher transparency of the water column.

### Microcystin concentration in the Nyanza Gulf

Coinciding with eutrophication, a regular MC occurrence was observed. MC concentrations recorded in Kisumu Bay and NG2, NG3 were among the highest from Lake Victoria that had ever been reported (Sekadende et al., 2005; Okello et al., 2010a, b; Haande et al., 2011). The highest MC concentrations were recorded between November and March coinciding with the wet season when rainfall and nutrient enrichment from the catchment increased. From the highly significant relationship between *Microcystis* and MC concentrations, it must be concluded that *Microcystis* is the major MC producer while the contribution of *Anabaena* is of minor importance. This conclusion corresponds to recent results on cyanobacterial blooms in Uganda (Okello et al., 2010a, b). Genetic studies (Okello et al., 2010a) demonstrated that, in samples from Uganda, the genotypes encoding MC synthesis assigned to *Microcystis* consistently occurred, while those assigned to *Anabaena* were never detected.

Consequently, *Microcystis* blooms persisting in large parts of the Nyanza Gulf may result in a risk of MC contamination of drinking water and of the food chain. An intoxication incident in Caruaru, Brazil in 1996 involving blooms of *M. panniformis* and *M. protocystis* resulted in fatalities of several dialysis patients (Komárek et al., 2001). A recent survey on the MC exposure risk from Ugandan lakes revealed that >50% of the WHO lifetime tolerable daily intake (TDI) guideline stems from the consumption of (untreated) drinking water (Poste et al., 2011). According to Reynolds (2006), even higher phytoplankton biomass (biovolume >10 mm^3^ l^−1^) could be predicted from the presently observed nutrient concentrations in the Nyanza Gulf in the near future. Strategies of dealing with MCs from lake water used for the drinking water supply should involve a regular monitoring of the cell numbers of toxigenic cyanobacteria in the raw water. When *Microcystis* cells pass a certain threshold (>2 × 10^3^ cells ml^−1^, Chorus & Bartram, 1999), additional treatment steps would be necessary, including flocculation and ozonation to remove the particles, followed by sand filtration or activated carbon filtration for removing dissolved MCs and other toxic compounds. Particularly sand filtration has been shown to be efficient in removing MCs from raw water (Chorus & Bartram, 1999). Nevertheless, raising public awareness through the media has to be fostered. Since, during dry periods, alternative water sources are scarce, the installation of in-situ/household biosand filtration units (Cronberg & Annadotter, 2006) that can be used for water purification should be considered.

## Electronic supplementary material

Below is the link to the electronic supplementary material.

Supplementary material 1 (PDF 32 kb)
