# Supplementary material for: Spatial variation of phytoplankton composition, biovolume, and resulting microcystin concentrations in the Nyanza Gulf (Lake Victoria, Kenya)
Source: Hydrobiologia. 2012 Mar 14;691(1):109–22. doi: 10.1007/s10750-012-1062-8 (PMC3968937; doi:10.1007/s10750-012-1062-8)
Supplement: Supplementary file 1 — Supplementary material 1 (PDF 32 kb) [file 10750_2012_1062_MOESM1_ESM.pdf]

Suppl. Table 1: List of phytoplankton species part of genera contributing >5% to total phytoplankton biovolume recorded at sampling points NG1-4 and KO5 (Fig. 2, 3).

Cyanobacteria:
Anabaena cf. discoidea Schmidle
Anabaena sp.
Anabaena cf. spiroides Klebahn
Cylindrospermopis africana Komárek & Kling
Merismopedia africana Komárek & Cronberg
Merismopedia tenuissima Lemmermann
Merismopedia glauca (Ehrenberg) Nägeli
Merismopedia punctata Meyen
Microcystis panniformis Komárek
Microcystis protocystis Crow
Microcystis botrys Teiling
Microcystis wesenbergii Komárek
Planktolyngbya circumcreta G.S. West
Planktolyngbya contorta Lemmermann
Planktolyngbya limnetica Lemmerman
Planktolyngbya tallingii Komárek & Kling

Bacillariophyceae:
Aulacoseira ambigua (Grunow) O. Müller
Aulacoseira granulata Ehrenberg
Cyclostephanos sp.
Cyclotella meneghiniana Kützing
Cyclotella sp.
Nitzschia dissipata (Kützing) Grunow
Nitzschia lacustris Hustedt
Nitzschia sp.
Nitzschia victoriae Sitoki & Rott (in prep.)
Nitzschia kavirondoensis Sitoki & Rott (in prep.)
Nitzschia mediocris Hustedt
Nitzschia palea (Kützing) W. Smith
Nitzschia recta Hantzsch
Synedra cunningtonii G.S. West
Synedra ulna (Nitzsch) Ehrenberg
Urosolenia victoriae (Schröder) Rott & Kling

Cryptophyta:
Cryptomonas sp.

Dinophyta:
Glenodinium benardinense Chodat & Zender
Glenodinium sp.

Euglenophyeae:
Euglena acus Ehrenberg

Euglena viridis Ehrenberg

Chlorococcales:
Ankistrodesmus falcatus (Corda) Ralfs
Ankistrodesmus fusiformis Corda
Ankyra judayi Corda
Coelastrum cambricum W. Archer
Coelastrum indicum Turner
Coelastrum microporum var. microporum Nägeli
Coelastrum pulchrum var. *pulchrum* Schmidle
Oocystis lacustris Chodat
Oocystis marssonii Lemmermann
Oocystis parva West et G.S. West
Oocystis tainoensis (Komárek) F. Hindák
Pediastrum boryanum (Turpin) Meneghini
Pediastrum duplex Meyen
Pediastrum tetras (Ehrenberg) Ralfs
Scenedesmus sp.
Selenestrum sp.

Zygnematophyceae:
Staurastrum gracile var. nyansae (Ralfs) G.S. West
Staurastrum planktonicum Teiling
Staurastrum limneticum Schmidle
